# Organoids in image-based phenotypic chemical screens

**DOI:** 10.1038/s12276-021-00641-8

**Published:** 2021-10-18

**Authors:** Ilya Lukonin, Marietta Zinner, Prisca Liberali

**Affiliations:** 1 Friedrich Miescher Institute for Biomedical Research (FMI), Maulbeerstrasse 66, 4058 Basel, Switzerland; 2 University of Basel, Petersplatz 1, 4001 Basel, Switzerland

**Keywords:** High-throughput screening, Drug delivery

## Abstract

Image-based phenotypic screening relies on the extraction of multivariate information from cells cultured under a large variety of conditions. Technical advances in high-throughput microscopy enable screening in increasingly complex and biologically relevant model systems. To this end, organoids hold great potential for high-content screening because they recapitulate many aspects of parent tissues and can be derived from patient material. However, screening is substantially more difficult in organoids than in classical cell lines from both technical and analytical standpoints. In this review, we present an overview of studies employing organoids for screening applications. We discuss the promises and challenges of small-molecule treatments in organoids and give practical advice on designing, running, and analyzing high-content organoid-based phenotypic screens.

## Mini-organs: toward successful image-based chemical screening

Advances in culture techniques and three-dimensional image analysis algorithms are needed for robust chemical screening of organoids, tiny three-dimensional versions of functioning organs that are derived from stem cells. Organoids can provide useful insights into potential drug targets, cellular behavior, and disease progression, but their complexity provides unique challenges for image-based screening and analyses. Prisca Liberali at the Friedrich Miescher Institute for Biomedical Research in Basel, Switzerland, and co-workers reviewed the current status of image-based chemical screening for organoid-based biological and drug research. Careful assay design and process optimization are required, and the volume of generated data can place strain on computational infrastructure. The team highlights the value of multiplexed imaging to improve efficiency, and suggests that neural networks and deep learning may help tackle the complexities of three-dimensional image segmentation analyses.

## Introduction

Since the first high-throughput imaging devices became commercially available ~20 years ago^1,2^, technological advances in automated microscopy have enabled researchers to perform image-based high-content screens with a large number of conditions in a relatively short amount of time. Such screens either use genetic approaches to perturb gene expression or use small molecules to modulate protein function^3^. Chemical compounds have advantages over genetic manipulations, especially in mammalian systems, due to their versatility: they can be used in systems that are not readily accessible by genetic techniques, they allow the inhibition and activation of protein function and the precise timing of treatment starting time and duration. Small-molecule-based screens have thus become the tool of choice for biological and drug discovery in academia and industry^4^. At the same time, the image-based readout of microscopy-based screens offers high information content, enabling phenotypic profiling. To fully utilize the potential of this experimental approach requires both a biologically relevant model system and a data-driven, unsupervised analysis strategy. In the following sections, we give practical advice with literature examples on the application of complex cellular systems, namely, organoids, in image-based high-content chemical screening, covering the main pillars of assay design, data acquisition, and analysis.

### Target-based and phenotype-driven screening pipelines

There are two main approaches for designing chemical screening assays: either based on perturbing the activity of a defined molecular target (target-based screening) or based on a known target phenotype (phenotype-driven screening). The target-based approach aims to identify compounds that affect the target in the desired way, so-called “hits” or “leads,” from a large library of candidates^5^. Advances in assay automation, particularly liquid handling and automated microscopy, have enabled high-throughput screens (HTS) for libraries ranging up to millions of chemicals. To leverage such massive assays, HTS trades readout complexity for high coverage: single-endpoint measurements such as cell viability^6^, proliferation^7^, or single gene reporter assays^8^ are typically used for hit selection. High-content screening (HCS), on the other hand, is a phenotype-based approach that relies on the extraction of phenotypic data. It captures information that is not available with classical high-throughput methods (such as target-unrelated readouts, cytotoxicity, protein localization, and others^9,10^) and generally aims at obtaining a comprehensive picture of the system.

High-throughput high-content imaging assays are at the interface of both approaches, enabling fully automated primary screening of large-compound^11^ and siRNA^12–14^ collections with high resolution and multiparametric readouts, revealing more detail about the target of interest and effects on general cell morphology. The pioneering technologies in this field were termed cell-based phenotypic assays^11,15,16^ and aimed at the characterization of the screened compounds in cellular contexts. Furthermore, whereas classical HTS readouts are well based, high-content analysis often relies on features extracted from single cells to unmask pleiotropic phenotypes and cell-to-cell variability in drug response^17,18^. HCS assays have thus earned their place in both the scientific discovery process and the modern drug development pipeline, illustrating the importance of complex, cell-based model systems coupled with multiparametric, phenotypic readouts^2,19^.

In the last decade, organoids and three-dimensional (3D) organotypic systems^20^ have become widely available to researchers. These complex structures recapitulate the architecture and function of in vivo organs and tissues and develop from stem cells or organ-specific progenitors in a self-organizing process^21–24^. Currently, a wide selection of tissues has been recapitulated in vitro with the help of organoid systems, and the list is continually growing. Naturally, scientists now aim to use organoids in chemical screening to leverage the power of both systems for biological discoveries.

### Adult and pluripotent stem-cell (PSC)-derived organoids

Organoids can be divided into two categories depending on the cells used to generate them: PSCs, including embryonic and induced PSCs, and adult stem cells (ASCs). The generation of organoids from PSCs recapitulates the sequence of events of embryonic development by exposing them to specific combinations and concentrations of morphogens, allowing the patterning of germ layers and, subsequently, tissues and organs. PSC-derived organoids include optic cup^25^, intestinal^26^, cerebral^27^, kidney^28^, thyroid^29^, lung^30^, and retinal^31^ organoids and others. Although they hold great potential, are used to generate a wide array of tissues, and can do so in a patient-specific context, the resulting organoids resemble embryonic rather than adult tissues^23,25,32^.

In contrast, ASC-derived organoids do not recapitulate developmental steps; rather, the regenerative capacity of parent tissues is utilized by the dissociation of biopsies and the subsequent culture of derived cells in artificial extracellular matrices. These include small intestinal^33^, colon^34^, lung^35^, mammary^36^ and salivary gland^37^, and pancreatic^38^ organoids and others. Consistently, the growth conditions for ASC-derived organoids typically include factors that control tissue repair or homeostasis, and the resulting structures are more mature than PSC-derived organoids^22^. However, the composition of culture media with respect to added growth factors and compounds often favors proliferation and culture expansion over cellular differentiation, which often limits the phenotypic heterogeneity that can be achieved. ASC-derived organoid systems usually mimic tissues that either have a high cell turnover rate or are capable of regeneration, such as the small intestine, stomach, and lung.

### Organoid model systems in chemical screening

Whereas target-based HTS assays can use simple readouts^39^ and minimalistic model systems^40^ because of their focus on perturbing target activity, phenotype-driven approaches require complex model systems with high disease or tissue relevance. Increasing the depth of compound profiling with phenotypic resolution thus goes hand-in-hand with increasing the physiological relevance of the screened system. Organoids are ideal candidates because they provide self-contained organotypic structures that mimic the cell-type composition and function of parent tissues with the major advantage that they are amenable to use in large screening assays^41^. In particular, organoids are accessible for imaging and standard liquid handling automation, meaning they can be used for standardized and well-established automated chemical screening pipelines. Thus, progressing from using isolated cells cultured in a monolayer to organoid structures in screening experiments is the next logical step. Combining organoids with small-molecule treatment offers several major advantages. First, organoids recapitulate individual steps of organ formation and disease onset, thus allowing the study of distinct developmental and disease stages. Second, small-molecule compounds can be added and removed from the culture medium at any given time, thus allowing targeted perturbation in a time-controlled fashion. In combination with a quantitative, data-driven, and unbiased analysis approach, chemical screening in organoids offers unprecedented insight into biological processes, delivering a wealth of data that can, nevertheless, be challenging to extract and analyze.

## Pillars of chemical screening in organoids

### Experimental question and system

For any biological assay, the first step lies in defining the process or target of interest. For a phenotypic screen, the model system should have the highest relevance to the tissue or disease of interest and yet be practical for the planned assay. For instance, when screening for compounds that affect a process specific to a given tissue, one should strive for a system that recapitulates its cell-type composition and morphology but also enables assay miniaturization and upscaling. A study employing this approach resulted in the identification of therapeutics preventing ZIKA virus infection by using stem-cell-derived brain organoids^42^. Assessing the drug response of a specific genotype is, on the other hand, best approached by screening patient-derived organoids with the goal of mapping patient-specific responses to available standard-of-care drugs. Of note, a thorough understanding of a process may result in a nontrivial choice of model system, which nevertheless is best suited for the question at hand. In a study pivotal for the organoid field, intestinal organoids were used to establish a diagnostic assay to predict the patient-specific response to standard-of-care drugs for treating pulmonary cystic fibrosis^43^. Phenotypes can be analyzed at the organoid level to answer broader biological questions: multivariate phenotypes of 400,000 intestinal organoids were recently used to systematically map functional interactions during organoid development and identify key players in intestinal regeneration^41^.

As discussed above, higher biological relevance can ultimately deliver novel biological insight and translate to better quality leads for the drug discovery pipeline, yet the assay has to be feasible in terms of cost and technology. In the next sections, we will focus on the underlying concepts and enabling technologies employed in organoid system screening.

### Assay development

**Fig. 1:**
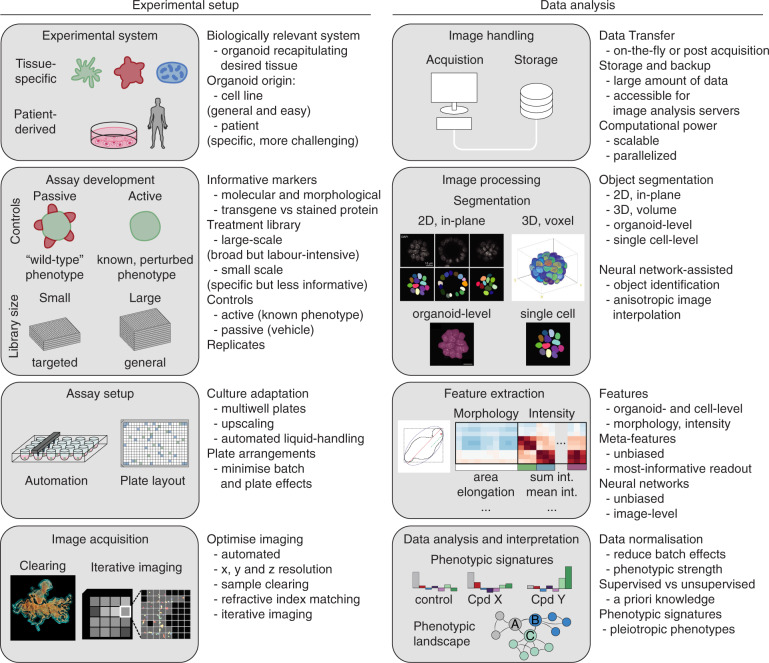
Overview of the screening process in organoids. A screen consists of experimental setup and performance and subsequently the analysis of the generated data. Both aspects include several steps with specific options or challenges. For a successful screen, these need to be evaluated and optimized in advance. Importantly, every decision in the assay setup, including organoid system, marker selection, type of controls, number of replicates, and imaging resolution, needs to be reconciled with steps in the data analysis process, including data handling, object segmentation, feature extraction approach, data normalization and interpretation, and vice versa. Figures are adapted from refs. ^41,52,57^.

**Fig. 2:**
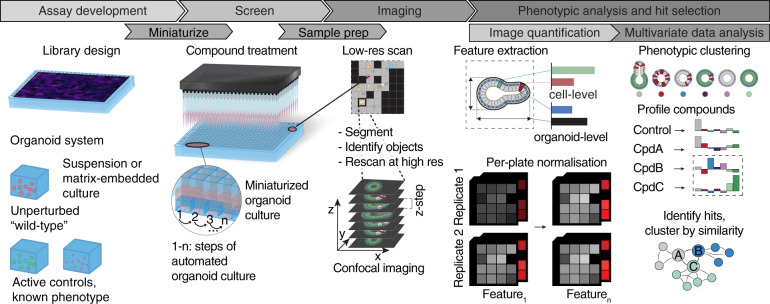
Design of the assay and analysis pipeline. For any organoid system, the culture protocol must be miniaturized to 96-, 384-, or 1536-well plate format and automated. At the same time, active controls that induce a measurable phenotype must be identified and incorporated into the screening library. The measurement relies on efficient yet high-quality imaging, for instance, employing iterative imaging approaches and generating images that are then used to extract cell- and organoid-level features. To make the data cross-comparable, plate normalization should be applied to minimize systematic variance between individual plates. The normalized features can then be used to cluster individual organoids by phenotypic similarity and profile screened conditions, assessing, for instance, the frequencies at which the identified phenotypes occur. Ultimately, multivariate analysis serves to select hits from the screen that can, in turn, be used to infer system-level properties, such as functional interactions. Figures are adapted from refs. ^41,83^.

After defining the biological question and identifying a suitable organoid system, one needs to design an experimental assay, which often involves multiple rounds of protocol optimization. Furthermore, this process demands fundamental knowledge of the biology and culture conditions of the chosen model system to decide on the screening setup. In the following section, we outline considerations in planning the experimental setup (Fig. 1) and give recommendations for individual steps of assay development, imaging, and data analysis (summarized in Fig. 2).

#### Marker selection

Selecting informative markers is a crucial step of assay design and strongly influences how a screen can be interpreted. Traditionally, dyes that stain the DNA content, including DAPI and Hoechst, are used to identify cell nuclei. Staining of a cell’s protein content (e.g., CellTrace), cytoskeleton (e.g., phalloidin), or membrane (e.g., CellMask) can be included to aid cell segmentation. Additional markers largely depend on the scientific question and may include markers of cell type, subcellular structures, or cellular processes, and identification of these markers is achieved either by immunofluorescent staining or by fluorescent tagging of the protein of interest. Importantly, one needs to verify both the correct labeling (antibody specificity in the case of staining; target protein functionality and stability and timely fluorescent protein activity in the case of tagging) of the marker and smooth performance of the labeling procedure in the case of immunofluorescent staining prior to conducting the screen. Another factor to consider is the cost of the resulting assay, whereas fluorescent reporters do not carry additional cost, they might lack signal strength, reducing the assay sensitivity. Antibodies, on the other hand, often offer superior signal and improved flexibility for combining wavelengths but increase the cost massively, especially for larger screens.

#### Small to large scale

The scale of a screen crucially influences several steps down the line of assay development and thus needs to be considered thoroughly. Genome-wide or large-scale screens offer the advantage of broad coverage and require less prior knowledge of the biological system. However, they also present a major cost factor due to increased system-specific culturing (specialized media, extracellular matrix components, and culture plates needed for organoid culture) and readout (increased use of immunofluorescence reagents and imaging time) requirements. Furthermore, the larger the scale is, the greater are the requirements for specialized equipment for handling large number of plates, time for sample preparation, imaging and data storage space. If a specific and defined question needs to be addressed, one may consider the advantages of a small-scale approach. A defined library targeting genes expressed at a certain developmental stage, in a specific organ, in a disease of interest or belonging to a relevant group of signaling pathways might be sufficient to shed light on the biological process while reducing costs and workload.

#### Treatment time frame and screen endpoint

The decision on when and for how long to apply screened conditions and at what point to read out results depends largely on the scientific question and the prior knowledge of the biological system. Especially for PSC-derived organoids, which follow the development of the organ from an early stage, treatment at an early progenitor stage compared to a more mature stage elicits drastically different responses. Moreover, ASC-derived organoids usually undergo different phases recapitulating regenerative and homeostatic conditions. In addition, the treatment duration needs to be considered carefully. Treatment throughout the formation of the organoid allows assessment of the influence of the perturbation on its complete development or the onset of a disease phenotype. However, additional validation experiments are usually necessary to understand which specific step is affected. To study a particular event during organoid formation, such as the emergence of a cell type or morphogenetic change, perturbation should be applied around the time when this event occurs.

Finally, if the screen is not read out continuously with time-lapse imaging^44^, an endpoint needs to be determined. Screens can be terminated when the organoid reaches a mature state, yet this also goes hand-in-hand with increased costs and might not be necessary when an intermediate step is to be studied.

#### Controls, plate arrangement, and replicates

For every scientific experiment, measures need to be taken to ensure the quality and statistical relevance of the screen, particularly when considering a larger scale. Controls must always be included in the assay, ideally in every plate, for quality control and the determination of the phenotypic feature space. These should preferably include both passive and active controls, whereby passive controls, which include no active substance (for instance, treatment with the vehicle alone), are used to assess reproducibility and to normalize the data, compensating for potential batch effects. Furthermore, one needs to determine a priori if and how the passive control might affect organoid formation and growth. Active controls, on the other hand, elicit a desired phenotype and are used to assess the validity of the screen and serve as comparison to identified hits, but they are not always readily available.

The number of passive control wells to include depends largely on the downstream statistical analysis: if normalizing results to passive controls alone (when a phenotypic effect is expected from most treatment conditions), more replicates of the control condition, usually ~10%, need to be included than if the whole plate will be used for normalization (when a phenotypic effect is expected from only a small number of treatment conditions). In general, as many control wells as possible and practical should be included to facilitate downstream analysis.

When using multiwell plates, plate arrangement of the controls is crucial to avoid the introduction of bias^45^. Experiments in multiwell plates are susceptible to plate effects^46^ that cause different behaviors in organoids in the edge wells due to faster evaporation of the medium. If a potential plate effect cannot be excluded, edge wells may be avoided. Controls should be distributed across the plate in a randomized order to detect and normalize artifacts such as edge effects, liquid dispensation differences, or thermal gradients^47^.

Replicates are used to rule out false positives and false negatives and to determine the variability within a perturbation. They can range from individual organoids within a well to replicate wells within the same screening run or independent screening runs or can stem from parallel screening of different cell lines, mouse, or patient origins. Ideally, a screen has two or more independent replicates (two screens conducted after each other or with different material) and several dependent replicates (organoids treated with the same compound within a run). However, the number of the latter depends strongly on how easily organoids can be obtained and might range from ten to several hundred. The statistical benefit of a larger number of replicates needs to be balanced against increased costs and workload and depends largely on the phenotypic consistency of the organoid system, which can be determined using positive controls.

#### Culture system setup

HTS and HCS employ automated culture, sample preparation, and readout to deal with the large number of samples. Most original organoid protocols, however, are manual and do not employ multiwell plates. Protocol adaptation and automation include simple transfer into the automation system^48^, upscaling^41,49^, transfer of already formed organoids into the screening plates^45^, or changes to the protocol that allow simpler automation^50^. The use of automation systems brings additional benefits, such as reduction of human bias and, thus, improved reproducibility and consistency of organoid cultures. It should, however, be assessed whether changes in the culture and assay format alter the organoid behavior.

#### Optimization and pilot screen

For the success of a screen, optimization and preparation are key. Each step—from organoid culture^51^ to perturbation administration, sample preparation, and, ultimately, screen readout—needs to be thoroughly tested. It is advisable to perform either a small pilot screen or a dry run, such as performing all steps with water and a small number of plates. This allows to test for an error-free process and creates a test dataset that can be used to set up the analysis pipeline.

### Data acquisition

The power of screening assays lies in systematic comparison of numerous conditions, minimizing experimental and technical variance. High-content imaging offers an unmatched combination of throughput and information density: organoids cultured in thousands of arrayed conditions^41^ can be imaged at high resolution to extract single-cell features^52^, describing observed phenotypes in depth.

To date, a variety of organoid systems have been studied with HCS to extract different kinds of information, relying mostly on immunofluorescence or reporter expression at the terminal time point^41,42,49,51^. For practical reasons, however, the number of readouts is often limited, and one of the crucial limiting factors is imaging. Most imaging systems offer up to five independent fluorophores per acquisition, effectively setting a cap on the number of readouts. Multiplexed imaging offers a cost-efficient solution to increase the information density by repeated imaging of the same sample probed for different readouts. One of the methods best fitted for this application is antibody elution: state-of-the-art protocols offer means to image up to forty antibody-bound epitopes^53^ in the same sample using common imaging devices and fluorophores. The benefits of multiplexed imaging for organoid systems include not only more thorough phenotypic profiling but also improved cost efficiency, as additional information can be extracted from the same sample. Another method making use of multiparametric profiling and multivariate phenotypes is cell painting^54^, a profiling assay describing every single cell with eight subcellular components. For poorly defined targets, this could be the only option: hits from such a screen would include conditions where for instance, a wild-type phenotype is restored in an organoid disease model. While the original protocol^55^ uses generic cell features such as cell morphology and nuclear and organelle structure, for organoids, this could be integrated with or replaced by cell-type composition.

As described above, imaging can deliver a comprehensive description of the phenotypes observed under the screened conditions. However, to obtain high-quality imaging data, one needs to consider the challenges presented by 3D model systems. The most appropriate imaging method for organoids is confocal microscopy, as it allows optical sectioning of the sample. For a screen consisting of several multiwell plates, however, confocal microscopy can result in extremely large amounts of imaging data: imaging just a single well in a 384-well plate with ten confocal planes and four readouts at high magnification can result in up to 3000 images, making data collection, storage, and processing challenging. This can be circumvented by selecting acquisition regions with iterative imaging: the sample is first scanned at low resolution to identify the positions of organoids, which are then reimaged at the desired higher magnification. This approach can increase the imaging throughput manifold while simultaneously reducing the amount of imaging data produced without any fidelity loss^41^.

An important factor influencing the confocal imaging setup is the resolution in the third dimension, the z-step: it requires an initial decision on whether the images produced will be used to reconstruct the organoid in 3D. For medium- and high-throughput assays, this approach would likely be impractical due to the imaging time per sample and amount of data generated per condition. An alternative solution is to subsample the third dimension of the organoid, setting the z-step to approximate cell size to ensure that all cells comprising the organoids are sampled. A further step in simplifying the readout and reducing the data amount is the generation of intensity projections, effectively reducing every 3D organoid to a 2D projected image that still contains the information necessary for phenotypic profiling.

Another challenge related to the third dimension is the sheer size of organoids: they can consist of thousands of individual cells spanning several hundreds of microns. To ensure imaging quality in the upper *z*-planes, it is often necessary to use sample clearing and/or refractive index matching, which increase the penetration depth and ultimately facilitate imaging of the entire organoid^52,56^.

Producing a high number of images places a strain on the computation infrastructure and requires a well-planned data lifecycle: before starting an image-based screen, one should try to estimate the storage space required for the dataset, ideally using a pilot assay with an equivalent sample. Furthermore, once acquired, images need to be transferred to storage that are accessible by the servers running the image analysis software.

#### Object segmentation

Image segmentation is the foundation of quantitative image analysis, converting intensity images into labeled maps used for downstream feature extraction. As simple as the task might sound, it can become increasingly difficult for 3D systems due to a plethora of technical and biological factors. In the last decade, 2D single-cell segmentation has become a widespread and almost trivial task, aided by an arsenal of automated algorithms. Most commonly used methods of foreground–background thresholding, however, perform poorly on organoid datasets due to signal inhomogeneity in the third dimension. Whereas the correction of illumination in 2D images is widespread and well established, compensating for signal decay in 3D is far less trivial: light scattering occurring due to deeper penetration into the sample can be highly nonlinear. This problem can be addressed experimentally, as discussed above, but nonetheless often requires adapting segmentation algorithms. Deep convolutional neural networks (DCNNs) offer an efficient and robust solution to this problem. The DCNN workflow requires a curated ground truth dataset used for training the artificial neural network to recognize desired objects, such as nuclei, in images of varying quality^57,58^. Given the unreliable results of threshold-based segmentation in organoid images, the overhead of creating a training dataset is overshadowed by the performance of neural networks. Another field of application for deep learning methods is image interpolation: as discussed above, generating isotropic images for a high number of organoids is not technically feasible but can be achieved by DCNN-assisted image interpolation. Algorithms such as CARE^59^ allow interpolating planes in subsampled 3D datasets, paving the way for single-cell-level organoid screens at a large scale.

#### Enhancing the depth of phenotypic profiling

A non-image-based method that delivers a wealth of information at single-cell resolution is single-cell RNA sequencing. It is, however, technically and financially infeasible to profile thousands of arrayed conditions. Furthermore, despite providing a comprehensive picture of gene expression, single-cell sequencing does not deliver information on individual organoid phenotypes and lacks the organoid context of the sequenced cells. Recent protocols try to fill this gap: single organoids can be first imaged to extract phenotypes and then sequenced to profile gene expression changes^60,61^. Similarly, in situ sequencing approaches can be used in organoids, enabling the transcriptomic profiling of intact organoid structures^62^. While still in its infancy, this methodology opens exciting new possibilities for organoid research.

### Feature extraction

In multivariate image analysis, every object, such as a single cell or an organoid, is described with extracted features relating to either the morphology (e.g., size and shape) or the intensity of the measured channels. To quantitatively describe the observed phenotypes, one should select features with high information content and low technical variance. The first can be achieved by calculating the amount of information encoded by a given feature, whereas the latter can be done systematically by computing feature covariance matrices and eliminating highly redundant and noisy features^11^. An alternative approach to feature selection is the generation of metafeatures, such as principal components (PC), allowing the unbiased selection and integration of most informative readouts. It should be noted that metafeatures have limitations; for instance, PCs are not able to encode nonlinear interactions. Furthermore, metafeatures often lack interpretability, as they do not represent a single, intuitively understandable parameter. Recent work also proposes the use of DCNNs for unbiased image parametrization, delegating feature selection to the neural network algorithm^63^. This can be achieved by training DCNNs on images coming from positive and negative controls, learning image-level features and using the resulting network for the detection of non-wild-type phenotypes^64^. Similarly, machine learning methods, particularly deep learning, can be applied to detect disease phenotypes in patient-derived organoids, as reviewed extensively in refs. ^65,66^.

#### Cell- and organoid-level information

A key difference between data from 2D cell assays and data from organoids is in the levels of imaged object hierarchy: in the single-cell quantification of 2D cell assays, every cell is directly linked to the condition, i.e., the well, but the organization of organoid data is more complicated. In fact, organoid data feature an extra level of complexity: every cell can be assigned to a parent organoid, which in turn is assigned to the treatment condition^67^. The properties of individual cells composing the organoids give rise to emergent properties of the system, and cell-to-cell variability is a crucial driving factor for organoid development^57^. This reflects the complexity of the organoid system and requires a paradigm shift in addressing quantitative data at single-cell resolution. Depending on the question at hand and the number of organoids sampled per condition, single-cell data can either be used as-is or be converted to organoid-level descriptors, such as cell-type composition.

#### Data normalization

In a screening assay, data points typically come from individual plates, making well plates the experimental units. As outlined above, every plate should contain a sufficient number of controls to allow per-plate normalization. A frequently used normalization strategy is z-scoring, normalizing the difference between the measured value and the mean of the reference condition by the standard deviation of the reference. *Z*-score normalization results in cross-comparable values that also reflect the strength of the effect and whether the feature is increased or decreased in the observed condition. Due to the nature of the method, the appropriate reference population should always be identified. When normalizing to a control condition, an insufficient number of control replicates or high variance in a given feature would result in poor *z*-score values, masking the true extent of the phenotype. This is especially true for organoid models that contain several cell types and show a high degree of morphological variability. If normalization is performed to the mean of the entire plate, the majority of the objects should not exhibit a phenotype; similarly, there should be no bias in condition distribution between plates. For example, conditions resulting in reduced organoid size and viability should not be grouped on a single plate of a multiplate experiment. It should be noted that an entire arsenal of statistical tools is available for addressing cases where frequently used algorithms perform poorly^46,68^. Overall, normalization is crucial to enable cross-comparison of data points from individual batches of the assay, and the normalization strategy should be chosen according to the assay design and the data distributions obtained.

#### Quantitative phenotype description

Quality control is well defined for single-parameter HCS workflows through metrics such as z-prime^46^, which describes the dynamic range between positive and negative controls. For phenotypic screens, however, no universally applicable parameters exist. As phenotypes are often complex and described by differences in several readouts, clustering in multivariate feature space can be used to assign similarity classes. Depending on the a priori knowledge of the system, phenotypes can be classified either in a supervised or in an unsupervised, data-driven manner. For an assay with known phenotypic effects, for instance, rescue of the wild-type organoid phenotype, machine learning algorithms such as support vector machines or random forest classifiers can be used to distinguish between individual data points. Alternatively, phenotypic classification can be performed by unsupervised clustering of the entire dataset using software packages such as PhenoGraph^69^. Once classified, conditions can be ranked by the presence of phenotypes not observed in the controls and by depletion of the “wild-type” phenotype. Due to the variability observed in organoid systems, however, control conditions can present pleiotropic phenotypes, meaning that no single control phenotype can be assigned. To address this, one can generate phenotypic signatures for every condition, describing the abundance of all detected phenotypic classes. In this approach, differences in phenotypic distributions describe every condition, making it straightforward to identify conditions that differ from the controls^41^. Furthermore, comparing phenotypic signatures between replicates of the same condition can be used to assess reproducibility.

### What can we learn?

Since the first organoid system was established more than a decade ago^70^, a multitude of systems have followed and enabled scientific discoveries that otherwise would have been challenging or even unattainable. As scientists start to employ organoids in chemical screening, an even greater potential becomes accessible.

Screening in organoids is dominated by disease- and drug discovery-driven research. Recently, however, it has also been leveraged by basic science to further our understanding of fundamental biological processes. A factorial screening approach of small molecules in embryoid bodies led to the formulation of an ideal culture medium composition for the robust derivation of several embryonic lineages in in vitro structures, facilitating the study of early embryonic development^71^. A similar approach was taken to improve and increase the throughput of kidney organoid culture, which was subsequently used to screen the drug response of organoids modeling a kidney disease^51^. A deep understanding of how organoids and their organs of origin develop can also directly benefit the identification of disease treatments: an inhibitor of the retinoic acid signaling pathway identified in a high-content screen of intestinal organoid development improved tissue regeneration after irradiation in mice, providing a lead for the treatment of tissue damage caused by radiation cancer therapy^41^.

Undoubtedly, organoid screening provides immense potential for the drug development process, and the peak of this development will be when the first organoid-developed treatment receives approval. The possibility of generating organ-like structures from human cells not only reduces the amount of animal testing but also enables the modeling of human-specific diseases with cells originating from patients. Drug screens in organoids are currently used to identify potential leads, test their efficacy, and assess their toxicity^72,73^. The advantages of using organoids instead of a single cell type grown in a monolayer lie in their diverse cell-type composition, which unmasks potential side effects in cell types not specifically targeted by the drug, as well as in their 3D architecture, which recapitulates the correct drug uptake^74^. However, the greatest potential of organoids lies in their application in personalized medicine. The main challenge in finding a cure for malignancies such as cancer is their heterogeneities, meaning that tumors in the same organ in two patients do not necessarily share the same genetic mutations, and even cells within a single tumor differ in their genotype^75^. This makes treatment choice challenging and often results in relapse if not all tumor cells are eliminated by the treatment. Cancer organoids grown from a patient biopsy reflect the full scope of tumor diversity and are used to identify the most efficient drug or drug combination to treat a particular patient^76–78^. This significantly increases positive treatment outcomes and decreases potential adverse effects.

In summary, although chemical screening in organoids is still in its infancy, it has already been leveraged for diverse use cases from basic to applied sciences, enabling profound scientific insight.

## Outlook

Organoids have opened vast possibilities to study biological processes in health and disease in an organotypic setting. By combining this potential with chemical screening, scientists have a powerful tool at hand to increase both the speed and reliability of scientific discoveries. Since organoids capture much of the complexity of tissues and organs, including structural and functional composition, but are still amenable to experimental interference, they are a suitable system to study development and homeostasis as well as the manifestation of diseases. Further developments in the organoid field, such as cocultures with immune cells or different tissue types, will surely improve the biological relevance of organoid models and bring them closer to their in vivo counterparts. Additional technical advances in culture conditions, liquid handling machinery, and image analysis algorithms will allow us to increase the homogeneity and reproducibility of organoid protocols and screening approaches, enabling reliable and advanced conclusions from organoid chemical screening^79,80^. Furthermore, since they can be derived from iPSCs or ASCs of patient origin, organoids pave the way for precision and personalized medicine. The establishment of organoid biobanks facilitates the use of organoids in screening to better understand disease and for drug discovery, development, safety, and efficacy^81,82^. Simultaneously, as both high-throughput organoid culture and HCS approaches also become more accessible to scientists in academia, the insights we can gain in basic research by these means will certainly increase.

In summary, screening in organoids introduces a multitude of possibilities for translating scientific discoveries to patients’ quality of life. With the continuous improvement of organoid culture, a growing list of organoid models and technical advances in HCS, there is still more to come.
